# Nasal Patency in Sitting, Supine, and Prone Positions in Individuals with and without Allergic Rhinitis

**DOI:** 10.3390/life13051226

**Published:** 2023-05-22

**Authors:** Yun-Ting Wang, Yao-Te Tsai, Cheng-Ming Hsu, Ming-Shao Tsai, Hsin-Yi Tsai, Geng-He Chang

**Affiliations:** 1 Department of Otolaryngology—Head and Neck Surgery, Chang Gung Memorial Hospital, Chiayi 613, Taiwan; 2 Faculty of Medicine, College of Medicine, Chang Gung University, Taoyuan 333, Taiwan; 3 Graduate Institute of Clinical Medical Sciences, College of Medicine, Chang Gung University, Taoyuan 333, Taiwan; 4 Head and Neck Infection Treatment Center, Chang Gung Memorial Hospital, Chiayi 613, Taiwan

**Keywords:** nasal patency, position, allergic rhinitis

## Abstract

(1) Background: Physiological changes in nasal patency in response to posture contribute to sleep-related problems. Previously, we reported that the supine and prone positions cause a significant decrease in nasal patency in subjective and objective assessments of healthy individuals. Therefore, we conducted a study to evaluate the effect of posture on nasal patency in patients with allergic rhinitis (AR); (2) Methods: The present study comprised 30 patients diagnosed with AR and 30 healthy subjects without nasal disease (non-AR). Changes in nasal patency were evaluated in the sitting, supine, and prone positions. We used the visual analog scale to evaluate subjective nasal blockage. Acoustic rhinometry and endoscopy were used to objectively measure changes in nasal patency; (3) Results: In the non-AR group, the prone position had a significant effect on subjective nasal blockage compared with the sitting position, with significant decreases in the minimal cross-sectional area (mCSA) measured by acoustic rhinometry. Furthermore, endoscopy demonstrated a significantly increased inferior turbinate hypertrophy in the non-AR group. In the AR group, there was no statistical difference in subjective nasal blockage symptoms between the different positions. However, in objective examinations (acoustic rhinometry and endoscopy), the prone position showed significantly decreased nasal patency; (4) Conclusions: In patients with AR, subjective nasal blockage did not significantly increase in the supine or prone position. Endoscopy demonstrated increased inferior turbinate hypertrophy in supine and prone positions resulting in a significant reduction in nasal cavity mCSA, indicating an objective reduction in nasal patency.

## 1. Introduction

There are few studies on sleep position and nasal patency [1,2]. However, a deep understanding of the physiological changes to the nasal cavity caused by posture is an important cornerstone of the study of respiratory diseases related to sleep quality [3,4]. Previous studies have focused on the differences in the degree of nasal obstruction between the supine and sitting positions [5,6]. Our recent study examined the effect of the supine and prone positions on the degree of nasal obstruction in normal patients without nasal disease [7] and demonstrated that both positions cause increased nasal obstruction compared with the sitting position on subjective and objective examinations, with nasal patency being more affected by the prone position compared with the supine position. In light of the above findings, we aimed to further explore the effect of various postures on nasal cavity patency in patients with allergic rhinitis (AR) and provide a comparison with healthy individuals without AR. In addition to using questionnaires to subjectively assess symptoms and acoustic rhinometry for objective analyses, we used video-endoscopy to directly record and compare changes in the degree of inferior turbinate swelling in various postures [8]. The present study was designed to increase the understanding of the effects of posture-related physiological nasal changes in patients with AR.

## 2. Materials and Methods

### 2.1. Participants with and without Allergic Rhinitis

We screened patients for AR in an outpatient clinic after obtaining informed consent. A total of 30 patients with AR were enrolled in the experimental group of the present study. We used the visual analog scale (VAS) to assess rhinitis-related symptoms including nasal blockage, itchy nose, sneezing, and runny nose, with scores ranging from 0 to 100 [9,10]. The inclusion criteria were as follows: total VAS score for the four symptoms greater than 120 points; serum immunoglobulin (Ig) E level greater than 120 kU/L (ImmunoCAP^®^; Phadia AB, Uppsala, Sweden [11]); and a positive result for at least one inhaled allergen (i.e., grass pollen, house dust mites, animal dander, or mold) on multiple antigen simultaneous testing. The exclusion criteria were as follows: severe nasal septal deviation (NSD); previous sinonasal surgery, including inferior turbinoplasty, surgery for NSD, sinusitis surgery, nasal tumor resection, and skull base surgery; medical history of malignancy of the nasal cavity, sinus, or nasopharynx; endoscopic evidence of nasal polyps or sinusitis; current smoker; and upper respiratory tract infection within the last month. Participants receiving AR-related drugs, such as steroid nasal sprays or antihistamines, were required to stop using AR-related medications for at least two weeks before participating in the study.

The control group comprised 30 healthy volunteers without AR. The criteria for inclusion in the control group were VAS scores for the four main symptoms of AR of less than 30 points and a serum IgE level lower than 120 kU/L. The exclusion criteria were the same as those described above for patients with AR.

By using unpaired Student *t*-tests, the sample size of 52 was based on a power of 80%. Using a two-tailed *t*-test with *p* < 0.05, we estimated the sample size as 26 participants per group, with an allowance for a potential dropout rate of 20%.

The present study was reviewed and approved by the Institutional Review Board and was given the number 201801720B0C501.

### 2.2. Subjective and Objective Assessments of Sleeping Positions

**Figure 1:**
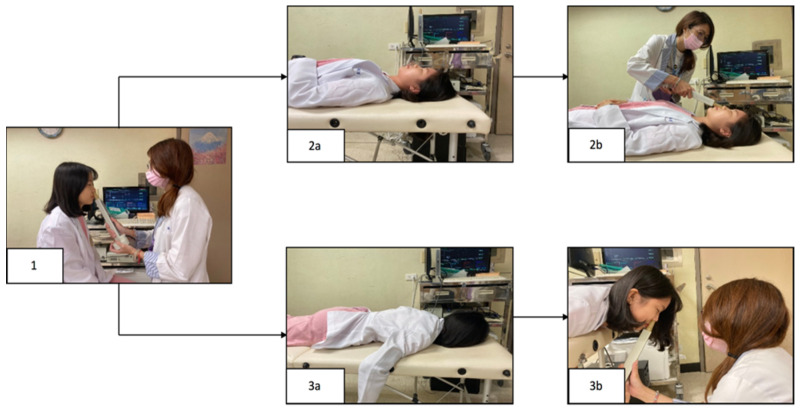
Flowchart of the postural examinations. Questionnaires, acoustic rhinometry, and endoscopy were performed with subjects in the sitting position (**1**). The subjects then changed to the supine position for 15 min (**2a**) and underwent the same subjective and objective examinations (**2b**). The subjects then changed to the prone position for the above three examinations (**3a**,**3b**).

**Figure 2:**
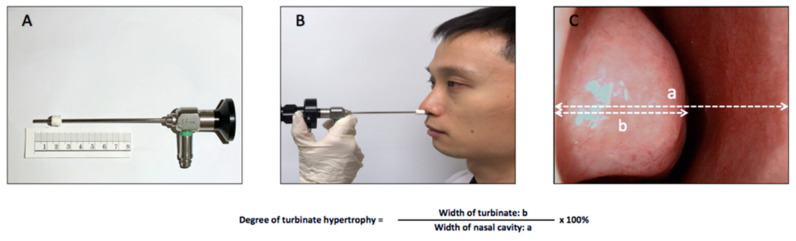
Measurement of nasal patency. (**A**) 1.7 cm mark behind the front end of a rigid endoscope. (**B**) The endoscope was inserted into the nasal cavity at a fixed depth to capture images. (**C**) The thickest part of the head of the inferior turbinate was used as the reference point with a horizontal line drawn. The width of the nasal cavity was set as “a”, the width of the inferior turbinate was set as “b”, and the degree of hypertrophy of the inferior turbinate was calculated by dividing “b” by “a” × 100 and presented as a percentage.

Three positions were assessed in this study: the sitting, supine, and prone positions [7] (Figure 1). Subjective nasal obstruction was assessed using VAS as the primary outcome and the objective results of acoustic rhinometry and endoscopy were secondary outcomes. We first asked subjects to sit and then used the VAS to assess the degree of subjective nasal blockage on a scale of 0 to 100. Next, minimal cross-sectional areas (mCSAs) of both nasal cavities were measured using acoustic rhinometry. The degree of turbinate hypertrophy was then recorded at a fixed distance using video-endoscopy [8] (Figure 2). Before the subjective and objective assessments were taken, the participants were asked to sit for 15 min. Then, the participants were asked to lie down for 15 min and VAS scoring, acoustic rhinometry, and endoscopy were again performed. Finally, the participants changed from the supine to prone position and rested for 15 min. The participants then completed the three subjective and objective examinations described above. All subjects completed the entire testing process in an environment with a humidity of 50–60% and a temperature of 25–26 °C.

The mCSAs of both nasal cavities were measured by acoustic rhinometry, with the sum used to compare objective nasal patency between different postures. In addition, we performed endoscopic examinations to directly observe changes in the inferior turbinate with different postures. The endoscopic examination method used was as described in our previous study [8]. A rigid endoscope (2.8 mm in diameter) was used with a mark 1.7 cm away from the front end (Figure 2). We placed the endoscope into the nasal cavity at a fixed distance (1.7 cm behind the nostril) for image recording. Serial images for each of the three positions were arranged and adjusted to appropriate positions and a horizontal line was drawn at the thickest part of the inferior turbinate as the reference point. The width of the inferior turbinate (distance “b” in Figure 2) was divided by the width of the nasal cavity (distance “a” in Figure 2). This value is presented as a percentage and represents the degree of hypertrophy of the inferior turbinate. Differences in the degree of hypertrophy of the inferior turbinate were compared between positions.

### 2.3. Statistical Analysis

Data on age, serum IgE levels, VAS scores, and mCSA were presented as mean ± standard deviation. Data were compared using the unpaired Student’s *t*-test, with *p*-values of less than 0.05 considered statistically significant. VAS scores, mCSAs, and endoscopy results for each of the three positions are presented as bar and dot graphs, with comparisons between groups performed using the paired Student’s *t*-test. * *p* < 0.05; ** *p* < 0.01; *** *p* < 0.0001.

## 3. Results

### 3.1. Study Population

A total of 60 subjects were included in the present study, including 30 patients with AR (15 women and 15 men; mean age 37.73 ± 13.40 years) and 30 healthy individuals without sinonasal disease (19 women and 11 men; mean age 31.13 ± 7.28 years). No significant differences in sex were observed between the two groups (*p* = 0.201) (Table 1). Of the four AR-related symptoms, the largest differences between the two groups were in nasal blockage (VAS score of AR vs. non-AR, 30.33 ± 26.58 vs. 7.67 ± 11.65, *p* < 0.001) and rhinorrhea (VAS score of AR vs. non-AR, 14.33 ± 21.44 vs. 4.33 ± 10.06, *p* = 0.026) in the sitting position. While the VAS scores for sneezing and itchy nose were higher in the AR group, the difference between the AR and non-AR groups did not reach statistical significance. In addition, mCSA measured by acoustic rhinometry did not significantly differ between the two groups in the sitting position (mCSA of AR vs. non-AR, 1.11 ± 0.29 vs. 1.00 ± 0.23 cm^2^, *p* = 0.269).

### 3.2. Subjective Assessment: Primary Outcome

**Figure 3:**
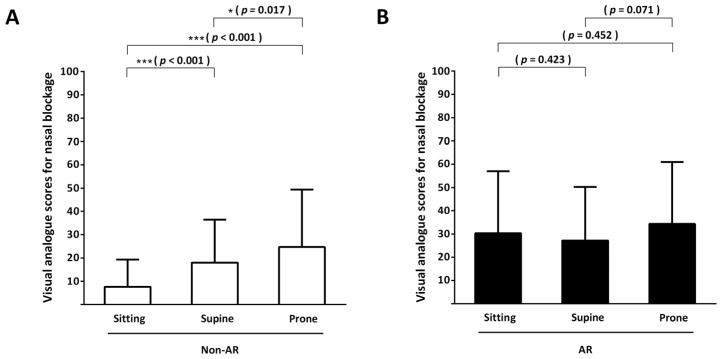
Visual analog scale. In healthy subjects without nasal disease (non-allergic rhinitis (AR) group) (**A**) and in patients with AR (**B**), the subjective assessment of nasal blockage in the sitting, supine, and prone positions was conducted using the visual analog scale with scores ranging from 0 to 100 points. * *p* < 0.05; *** *p* < 0.0001.

The VAS scores were used to assess subjective nasal blockage between the AR and non-AR groups in various positions. The results showed that in subjects without nasal disease, the supine position significantly increased the degree of nasal blockage compared with the sitting position (VAS score of supine vs. sitting positions in the non-AR group, 18.00 ± 18.46 vs. 7.67 ± 11.65, *p* < 0.001; mean score increased by 2.35 times), while the prone position was associated with a significantly higher degree of nasal blockage (VAS score of prone vs. sitting positions in the non-AR group, 24.67 ± 24.74 vs. 7.67 ± 11.65, *p* < 0.001; average VAS score increased by 3.22 times). The effect of the prone position on nasal blockage was more significant than that of the supine position (VAS score of prone vs. supine positions in the non-AR group (24.67 ± 24.74 vs. 18.00 ± 18.46, *p* = 0.017) (Figure 3A).

For patients with AR, both supine and prone positions did not significantly increase subjective nasal blockage (VAS score in AR patients: supine vs. sitting, 27.17 ± 23.11 vs. 30.33 ± 26.59, *p* = 0.423; prone vs. sitting, 34.33 ± 26.61 vs. 30.33 ± 26.59, *p* = 0.452) (Figure 3B).

### 3.3. Objective Assessments: Secondary Outcomes

#### 3.3.1. Acoustic Rhinometry

**Figure 4:**
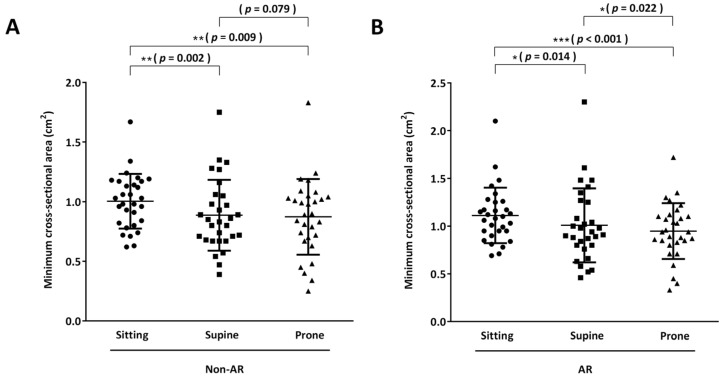
Acoustic rhinometry. Healthy subjects without nasal disease (non-allergic rhinitis (AR) group) (**A**) and patients with AR (**B**) underwent acoustic rhinometry to objectively evaluate nasal cavity patency in the sitting, supine, and prone positions. The results are presented as minimal cross-sectional area (cm^2^). * *p* < 0.05; ** *p* < 0.01; *** *p* < 0.0001.

Individual mCSAs for both nostrils in non-AR and AR subjects were measured using acoustic rhinometry and then summed for comparison. Results demonstrated that the supine and prone positions significantly reduced the mCSA in both non-AR and AR subjects compared with the sitting position (mCSA in the non-AR group: supine vs. sitting, 0.89 ± 0.30 vs. 1.00 ± 0.23, *p* = 0.002; prone vs. sitting, 0.87 ± 0.32 vs. 1.00 ± 0.23, *p* = 0.009; mCSA in the AR group: supine vs. sitting, 1.01 ± 0.39 vs. 1.11 ± 0.29, *p* = 0.014; prone vs. sitting, 0.95 ± 0.29 vs. 1.11 ± 0.29, *p* < 0.001; Figure 4A,B). Comparing the supine and prone positions, the prone position had lower mCSA in both non-AR and AR groups, but only the AR group had a statistically significant difference (mCSA in the non-AR group, prone vs. supine: 0.87 ± 0.32 vs. 0.89 ± 0.30, *p* = 0.079; mCSA in the AR group, prone vs. supine: 0.95 ± 0.29 vs. 1.01 ± 0.39, *p* = 0.022).

#### 3.3.2. Endoscopy

**Figure 5:**
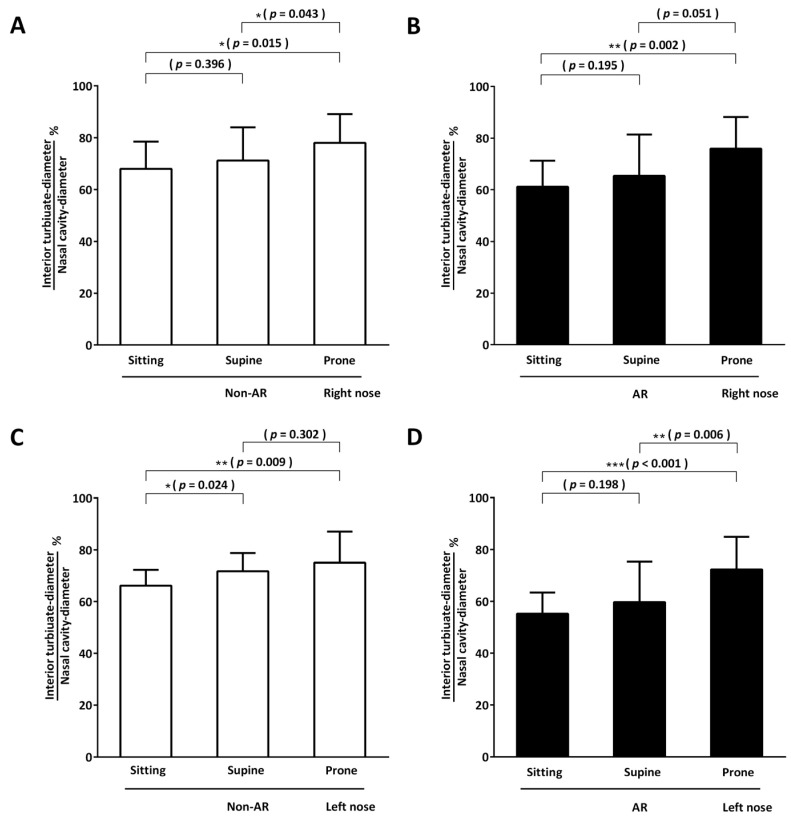
Endoscopic assessment. Healthy subjects without nasal disease (non-allergic rhinitis (AR) group) and patients with AR underwent video-endoscopy on both sides of the nasal cavity in the sitting, supine, and prone positions. The results are expressed as a percentage calculated by dividing the width of the inferior turbinate by that of the nasal cavity. * *p* < 0.05; ** *p* < 0.01; *** *p* < 0.0001.

We used endoscopy to detect changes in the degree of inferior turbinate hypertrophy in various postures (Figure 5A–D). We evaluated the left and right nasal cavities separately. The results showed that, regardless of the presence or absence of AR, the inferior turbinate had increased swelling in the supine position compared with the sitting position. This difference reached statistical significance for the left nostril of non-AR subjects (left nostril inferior turbinate diameter/nasal cavity diameter in the non-AR group, supine, 71.68% ± 7.05% vs. sitting, 66.15% ± 6.09%, *p* = 0.024). Other comparisons did not reach statistical significance. Inferior turbinate swelling was significantly increased in the prone position compared with the sitting position in both groups for both nostrils (inferior turbinate diameter/nasal cavity diameter for prone vs. sitting; non-AR group right nostril, 78.03% ± 11.13% vs. 67.98% ± 10.48%, *p* = 0.015; non-AR group left nostril, 75.02% ± 11.94% vs. 66.15% ± 6.09%, *p* = 0.009; AR group right nostril, 75.89% ± 12.28% vs. 61.12% ± 10.18%, *p* = 0.002; AR group left nostril, 72.23% ± 12.53% vs. 55.20% ± 8.21%, *p* < 0.001).

## 4. Discussion

We previously investigated the effects of the supine and prone positions on the degree of nasal blockage in healthy individuals without nasal disease [7]. Both subjective and objective assessments demonstrated that both supine and prone positions were associated with significantly reduced nasal cavity patency compared with the sitting position, and the prone position had a greater effect than the supine position. In the present study, the data in the control group (non-AR healthy participants) corroborated the results of our previous study. We also performed endoscopy in the present study to objectively assess changes in inferior turbinate swelling in response to various postures. These results also demonstrate that the head of the inferior turbinate was significantly more swollen in the supine and prone positions compared with the sitting position, indicating decreased nasal volume in the former two positions.

In the present study, we explored the effect of reduced nasal patency due to postural changes in patients with AR by comparing them with healthy individuals without nasal disease. We observed no significant difference in subjective nasal blockage between the sitting, supine, and prone positions; however, acoustic rhinometry and endoscopy demonstrated significantly decreased nasal patency in the prone position. We speculate that decreased nasal patency in patients with AR may be due to prolonged and compensated nasal blockage (Table 1). Therefore, the subjective nasal symptoms were not significantly affected in patients with AR despite the objective reduction in nasal patency. This finding implies that the degree of inferior turbinate swelling does not necessarily lead to symptoms of nasal blockage in patients with AR. Therefore, patients with AR may not be aware of the severity of nasal turbinate swelling and, without proper treatment, inflammation of the nasal mucosa may continue to increase, leading to further formation of nasal polyps and sinusitis [12].

As AR is a chronic disease with a high prevalence worldwide, it is likely to coexist with many respiratory diseases, including obstructive sleeping apnea (OSA) and sudden infant death syndrome (SIDS) [13]. An increased risk of developing SIDS has been reported in preterm compared with full-term infants, which has been posited to be related to the prone sleeping position [14]. Previous studies have demonstrated increased upper airway collapsibility in the prone position [15,16]. Therefore, the findings of the present study may provide an important basis for follow-up studies on the effect of supine and prone positions on nasal patency in patients with OSA and SIDS.

This study evaluated the effect of the supine and prone positions on nasal cavity patency. However, some people sleep in the lateral recumbent position. Thus, further studies are required to determine the effect of the lateral recumbent position on nasal patency, and increased knowledge of the physiological changes caused by this position may contribute to OSA studies.

One issue that warrants further investigation is the potential impact of comorbid conditions on the relationship between postural changes and nasal patency in patients with AR. Comorbidities, such as asthma, chronic sinusitis, or gastroesophageal reflux disease (GERD), can often coexist with AR and may affect nasal patency and congestion. Understanding the interplay between these comorbidities, postural changes, and nasal patency could provide valuable insights into the complexity of managing AR in patients with multiple medical conditions. For example, asthma is a common comorbidity in patients with AR, and bronchial hyperresponsiveness has been reported to be associated with increased nasal congestion. Investigating whether the effect of postural changes on nasal patency differs between AR patients with and without asthma could shed light on the possible interactions between respiratory function, nasal patency, and posture. In addition, GERD, which is known to be associated with the supine position and nighttime symptoms, can exacerbate AR symptoms by causing postnasal drip and nasal inflammation. It might be valuable to explore whether the effect of postural changes on nasal patency is more pronounced in AR patients with GERD, particularly during nighttime when reflux symptoms are more likely to occur. Identifying potential differences in the impact of postural changes on nasal patency among AR patients with various comorbidities could help develop more targeted treatment plans and interventions for these patients.

The topic worth studying is the potential role of medication use, including intranasal corticosteroids, antihistamines, and decongestants, in modulating the effect of postural changes on nasal patency in AR patients—pharmacological treatments for AR aim to reduce inflammation and relieve symptoms, including nasal congestion. However, the interaction between medication use, postural changes, and nasal patency has yet to be extensively explored. Understanding this relationship could help optimize medication use and improve symptom control in AR patients. For instance, the timing of medication administration concerning postural changes could potentially influence the effectiveness of treatment in alleviating nasal congestion. It might be worth examining whether administering medications at specific times relative to postural changes, such as before bedtime, could help minimize the impact of supine or prone positions on nasal patency in AR patients. Additionally, comparing the effect of different medications on postural changes and nasal patency might reveal specific drug classes that are more effective in mitigating the impact of posture on nasal congestion. This information could aid in developing tailored treatment regimens that consider the timing of medication administration and the choice of drug classes, in addition to postural changes, to improve symptom control in AR patients.

A deeper exploration of the influence of comorbid conditions and medication use on the relationship between postural changes and nasal patency in AR patients could help elucidate potential interactions and complexities in managing AR in patients with multiple medical conditions or under pharmacological treatment. A better understanding of these factors could ultimately contribute to developing more personalized and effective treatment strategies for AR patients, considering the presence of comorbidities and medication use, in addition to postural changes.

The influence of demographic factors, such as age and sex, on the effect of different positions on nasal patency in AR patients is worthy of being investigated further. Although our study did not find significant differences in sex between the AR and non-AR groups, further data stratification based on age and sex might reveal specific trends or differences in how different positions affect nasal patency in various subpopulations. A more in-depth analysis of the potential interactions between age, sex, and the effect of postural changes on nasal patency could lead to a better understanding of the pathophysiology of AR and help tailor more personalized treatment strategies for different patient populations. For instance, it is known that hormonal fluctuations during menstruation, pregnancy, or menopause can affect nasal congestion and mucosal edema in women. Therefore, it might be valuable to examine whether the effect of postural changes on nasal patency differs between premenopausal and postmenopausal women or during different stages of the menstrual cycle. Similarly, age-related changes in nasal structures and functions, such as reduced mucociliary clearance and nasal airflow, might influence the effect of different positions on nasal patency in older adults. Identifying potential differences in the impact of postural changes on nasal patency among various demographic subgroups could provide valuable insights for developing age- and sex-specific treatment recommendations and interventions for AR patients.

Another area of discussion is the potential role of environmental factors, such as allergen exposure, in modulating the effect of postural changes on nasal patency in AR patients. It is well-established that allergen exposure plays a crucial role in developing and exacerbating AR symptoms. However, the interaction between allergen exposure, postural changes, and nasal patency has yet to be thoroughly investigated. Understanding the relationship between these factors might help develop strategies to reduce the impact of allergen exposure on nasal congestion and improve patients’ quality of life. For example, patients with AR might be more sensitive to allergens during sleep due to prolonged exposure in specific positions, such as the prone or supine positions. It could be valuable to explore whether interventions targeting allergen reduction in the sleeping environment, such as using allergen-proof bedding materials or air purifiers, could help mitigate the effects of postural changes on nasal patency in AR patients. Additionally, investigating the temporal relationship between allergen exposure, postural changes, and nasal congestion could reveal valuable insights into the optimal timing of allergen avoidance measures or pharmacological treatments concerning sleep and postural changes.

Our study contributes to the broader understanding of nasal physiology and the development of novel interventions for AR and other respiratory disorders. As observed in our study, the discrepancy between the objective findings and subjective perception of nasal blockage in AR patients may be due to various factors, including neuroplasticity, neural adaptation, and altered sensory perception in chronic nasal congestion. Gaining a better understanding of the underlying mechanisms responsible for these phenomena could pave the way for developing new therapeutic approaches targeting nasal blockage’s sensory and neural aspects, in addition to the traditional treatment options focusing on inflammation and congestion. Furthermore, the potential influence of sleeping positions on nasal patency may lead to the development of new positional therapies or devices that aim to optimize nasal airflow during sleep. For instance, personalized positional pillows or wearable devices could be designed to promote specific sleeping positions or maintain an optimal head position that maximizes nasal patency for patients with AR or other respiratory disorders. These innovative interventions could complement existing treatment strategies, such as pharmacological therapies and behavioral modifications, to provide more comprehensive and effective management of these conditions.

Our study had several limitations. First, anatomical variations such as NSD may have influenced the effect of postural change on nasal patency. We excluded patients with significant NSD; however, computed tomography of the sinuses was not performed to correct for the effects of NSD. We are therefore considering the use of computed tomography in subsequent studies. Second, the objective analyses used in this study, including acoustic rhinometry and endoscopy, were performed by the same examiner with attempts made to ensure the instruments were used in the same position between subjects and postures. Acoustic rhinometry is a delicate examination that requires the nozzle to be placed tightly against the nostrils to avoid air leakage; however, excessive pressure could cause nasal valve deformation and affect the testing results. The difficulty of performing the test was even higher when the patients were examined in the prone position, and this would increase the risk of producing bias on examination. Third, in order to standardize the testing process, we fixed the testing sequence from sitting, supine, to prone positions; however this approach might cause bias which could be reduced by randomly positional changes.

## 5. Conclusions

Subjective and objective assessments in the present study demonstrated that the supine and prone positions cause increased nasal blockage compared with the sitting position in non-AR healthy individuals, and that the prone position has more influence on nasal patency than the prone position. In AR patients, although the objective findings were consistent with those for non-AR subjects, postural change had no significant effect on nasal blockage.

## Figures and Tables

**Table 1:** Characteristics of the study population.

Variable	Non-AR (*n* = 30)	AR (*n* = 30)	*p*-Value *
Sex			0.201
Women	19	15	
Men	11	15	
Age (years)	31.1 ± 7.3 #	37.7 ± 13.4	0.022
IgE (kU/L)	40.4 ± 24.9	247.6 ± 422.4	0.001
VAS (0–100)			
Nasal blockage	7.7 ± 11.7	30.3 ± 26.6	<0.001
Rhinorrhea	4.3 ± 10.1	14.3 ± 21.4	0.026
Sneezing	1.7 ± 4.6	6.0 ± 11.0	0.054
Itchy nose	7.0 ± 13.4	15.7 ± 23.0	0.080
mCSA (cm^2^)	1.0 ± 0.2	1.1 ± 0.3	0.269

Abbreviations: AR, allergic rhinitis; IgE, immunoglobulin E; mCSA, minimal cross-sectional area; VAS, visual analog scale. # mean ± standard deviation; * Chi-square and unpaired Student’s *t*-tests.

## Data Availability

Not applicable.
